# Supplementary material for: YTHDF1 Promotes Bladder Cancer Cell Proliferation via the METTL3/YTHDF1–RPN2–PI3K/AKT/mTOR Axis
Source: Int J Mol Sci. 2023 Apr 7;24(8):6905. doi: 10.3390/ijms24086905 (PMC10139185; doi:10.3390/ijms24086905)
Supplement: Supplementary file 1 [file ijms-24-06905-s001.zip › ijms-2269417-supplementary.pdf]

Table S1. Sequences of small interfering RNA or Primer sequences for quantitative real－time PCR

Designation	Genes	Genes	Sequences (5－3′)	Organism
Primer		YTHDF1	F：CACCCAGAGAACAAAAGGACA	Homo sapiens
			R：GTGAGGTATGGAATCGGAGGG	
		GAPDH	F：GGAGTCCACTGGCGTCTTCA	
			R：GTCATGAGTCCTTCCACGATACC	
		RPN2	F：ACTCACTCCTCACCAGACATTTG	
			R：TTGGGTTCTTCAAAGTGGCATCT	
		METTL3	F：GCAACGCATCATTCGGACAG	
			R：CTGGTTGAAGCCTTGGGGAT	
si-RNA		YTHDF1-siRNA-1	F：GGAUACAGUUCAUGACAAUTT	Homo sapiens
			R：AUUGUCAUGAACUGUAUCCTT	
		YTHDF1-siRNA-2	F：GCUCCAUUAAGUACUCCAUTT	
			R：AUGGAGUACUUAAUGGAGCTT	
		METTL3-si-RNA-1	F：GAGCCAGCCAAGAAAUCAATT	
			R：UUGAUUUCUUGGCUGGCUCTT	
		METTL3-si-RNA-2	F：GGAGAUCCUAGAGCUAUUATT	
			R：UAAUAGCUCUAGGAUCUCCTT	
		RPN2-si-RNA-1	F：GGCCACUGUUAAACUAGAATT	
			R：UUCUAGUUUAACAGUGGCCTT	
		RPN2-si-RNA-2	F：GGAUCGCCCUUUCACAAAUTT	
			R：AUUUGUGAAAGGGCGAUCCTT	

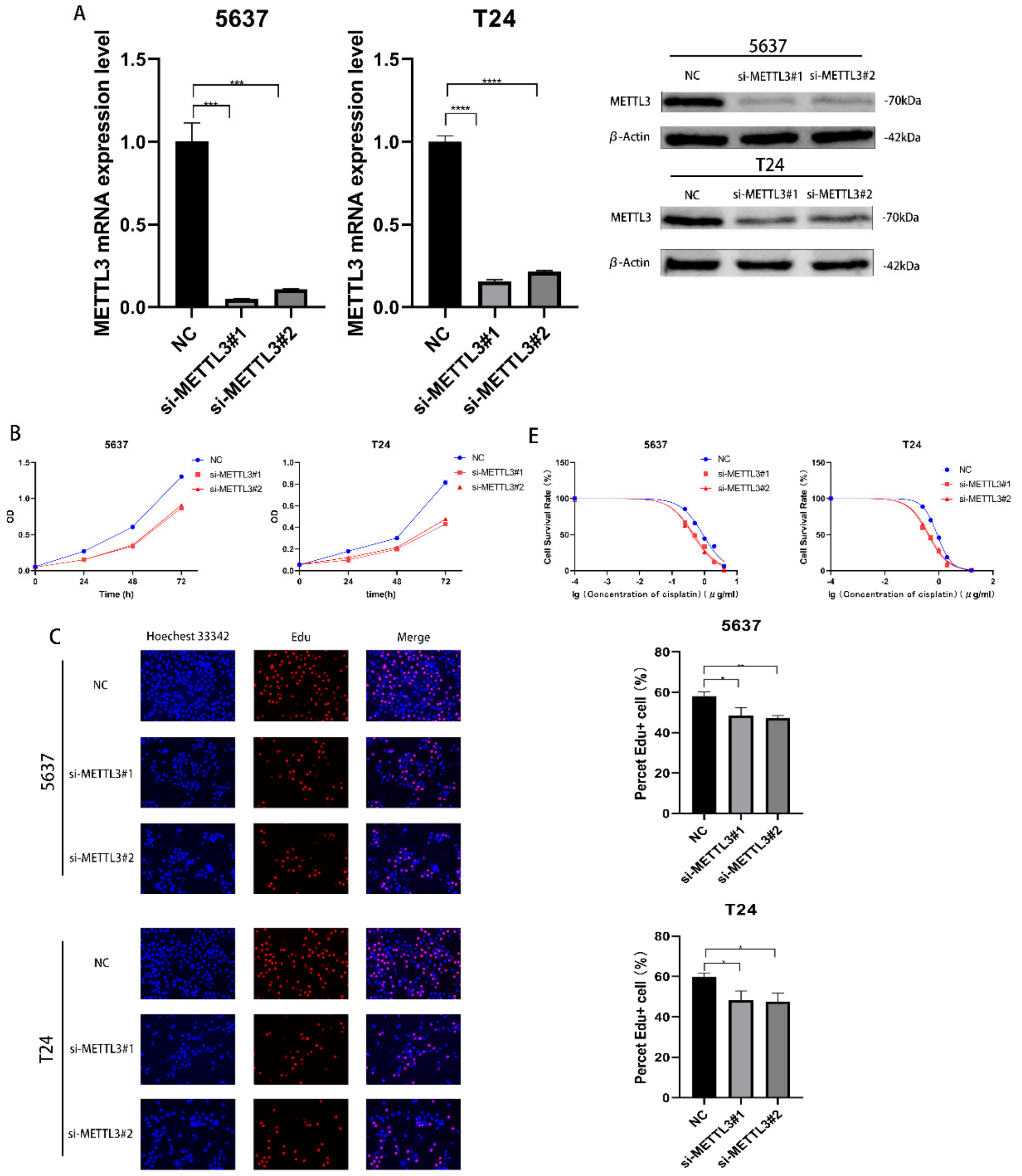

Figure S1. Effect of METTL3 on BLCA cell proliferation and cisplatin sensitivity. (A) Knockdown of METTL3 mRNA and protein levels in T24 and 5637 cells. (B-C) CCK-8 assay and EdU assay to verify the effect of METTL3 on BLCA cell proliferation and cisplatin sensitivity. (D) MTT assay to verify the effect of METTL3 on the cisplatin sensitivity of BLCA cells.

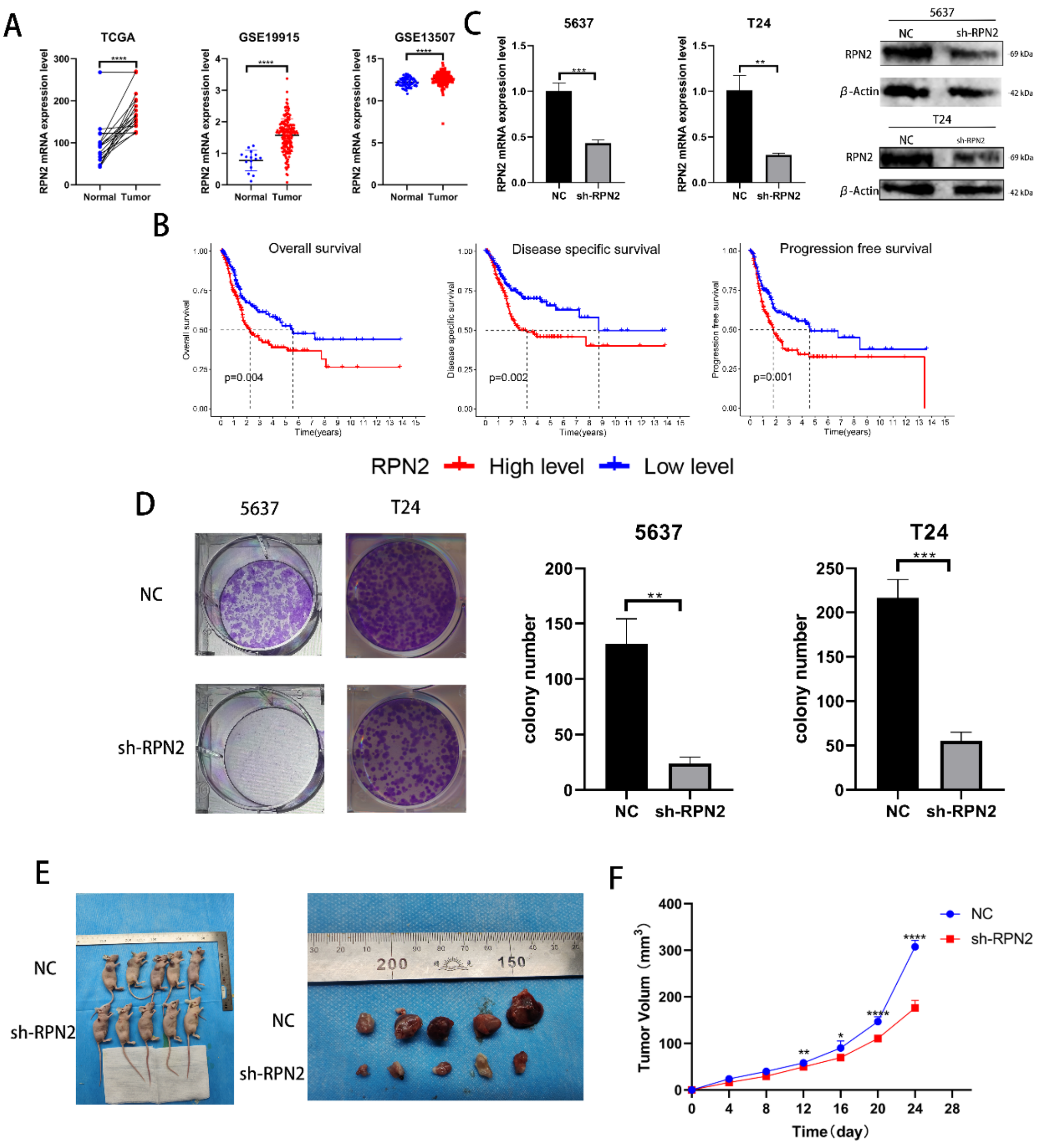

Figure S2. The relationship between RPN2 and clinical features and the effect of RPN2 on the proliferation of BLCA cells in vitro and in vivo. (A) Validation of RPN2 expression in the TCGA database, GSE1995 and GSE13507 datasets. (B) Effect of RPN2 expression in TCGA database with OS, DSS and PFI in BLCA patients. (C) YTHDF1 mRNA and protein levels were knocked down in T24 and 5637 cells. (D) Plate-cloning assay to verify the effect of RPN2 on the proliferative capacity of T24 and 5637 cells. (E-F) Knockdown of RPN2 expression to probe the effect on the T24 proliferative capacity in vivo.
